# Association of the *OPRM1* A118G polymorphism and Pavlovian-to-instrumental transfer: Clinical relevance for alcohol dependence

**DOI:** 10.1177/0269881121991992

**Published:** 2021-03-16

**Authors:** Miriam Sebold, Maria Garbusow, Deniz Cerci, Ke Chen, Christian Sommer, Quentin JM Huys, Stephan Nebe, Michael Rapp, Ilya M Veer, Ulrich S Zimmermann, Michael N Smolka, Henrik Walter, Andreas Heinz, Eva Friedel

**Affiliations:** 1 Department of Psychiatry and Psychotherapy, Charité – Universitätsmedizin Berlin, corporate member of Freie Universität Berlin, Humboldt-Universität zu Berlin, and Berlin Institute of Health, Berlin, Germany; 2 Department for Social and Preventive Medicine, University of Potsdam, Potsdam, Germany; 3 Klinik für Forensische Psychiatrie, Universitätsmedizin Rostock, Rostock, Germany; 4 Technical University of Dresden, Dresden, Germany; 5 Division of Psychiatry, University College London, London, UK; 6 Max Planck UCL Centre for Computational Psychiatry and Ageing Research, University College London, London, UK; 7 Department of Economics, University of Zurich, Zurich, Switzerland; 8 Department of Addiction Medicine and Psychotherapy, kbo Isar-Amper-Klinikum, Munich, Germany

**Keywords:** Alcohol dependence, learning, decision making, *OPRM1* A118G, opioid system

## Abstract

**Background:**

Pavlovian-to-instrumental transfer (PIT) quantifies the extent to which a stimulus that has been associated with reward or punishment alters operant behaviour. In alcohol dependence (AD), the PIT effect serves as a paradigmatic model of cue-induced relapse. Preclinical studies have suggested a critical role of the opioid system in modulating Pavlovian–instrumental interactions. The A118G polymorphism of the *OPRM1* gene affects opioid receptor availability and function. Furthermore, this polymorphism interacts with cue-induced approach behaviour and is a potential biomarker for pharmacological treatment response in AD. In this study, we tested whether the *OPRM1* polymorphism is associated with the PIT effect and relapse in AD.

**Methods:**

Using a PIT task, we examined three independent samples: young healthy subjects (*N* = 161), detoxified alcohol-dependent patients (*N* = 186) and age-matched healthy controls (*N* = 105). We used data from a larger study designed to assess the role of learning mechanisms in the development and maintenance of AD. Subjects were genotyped for the A118G (rs1799971) polymorphism of the *OPRM1* gene. Relapse was assessed after three months.

**Results:**

In all three samples, participants with the minor *OPRM1* G-Allele (G+ carriers) showed increased expression of the PIT effect in the absence of learning differences. Relapse was not associated with the *OPRM1* polymorphism. Instead, G+ carriers displaying increased PIT effects were particularly prone to relapse.

**Conclusion:**

These results support a role for the opioid system in incentive salience motivation. Furthermore, they inform a mechanistic model of aberrant salience processing and are in line with the pharmacological potential of opioid receptor targets in the treatment of AD.

## Introduction

Contextual stimuli are important modulators in the way we learn and can promote specific behaviours. One mechanism underlying contextual learning is the so-called Pavlovian-to-instrumental transfer (PIT). The PIT effect capture the influence of Pavlovian conditioned stimuli (CSs) on instrumental behaviour, with appetitive Pavlovian stimuli specifically promoting approach and reducing withdrawal, and aversive Pavlovian stimuli promoting withdrawal and reducing approach (Huys et al., 2011), thus reflecting a powerful mechanism affecting behavioural choices across humans (Talmi et al., 2008) and animals (Dickinson et al., 2000; O’Connor et al., 2010). Moreover, the PIT effect has been used as a quantification of incentive salience attribution, that is, the extent to which formerly neutral cues become attractive, themselves desired, and therefore ‘wanted’ (Huys et al., 2014; Meyer et al., 2012).

Crucially, incentive salience attribution is one prominent mechanism underlying several disorders of compulsivity, such as alcohol dependence (AD; Corbit and Janak, 2007) and other addictive disorders (LeBlanc et al., 2012). Also, interindividual differences in PIT have been associated with addiction vulnerability and maintenance. For instance, preclinical work suggests an association between the magnitude of PIT and addictive behaviour, such as compulsive alcohol drinking (Barker et al., 2012; Corbit and Janak, 2007). Preclinical studies have also consistently reported that non-drug-related (e.g. food or sucrose reward) CSs lead to increased responding during PIT in addicted animals (LeBlanc et al., 2013; Ostlund et al., 2014; Saddoris et al., 2011). Moreover, we have recently shown that the PIT effect in humans serves as a vulnerability marker for the development and maintenance of AD (Garbusow et al., 2014, 2019; Schad et al., 2019a; but see van Timmeren et al., 2020). The behavioural and neural correlates of PIT have been associated with relapse in AD (Garbusow et al., 2016; Sekutowicz et al., 2019; Sommer et al., 2020) and were predictive of future drinking behaviour in adolescents (Sekutowicz et al., 2019).

Although contemporary theories emphasise the involvement of the dopaminergic system in incentive salience, recent findings suggest the opioid system as another important player (Pecina and Berridge, 2013; van Steenbergen et al., 2019). The opioid system has been primarily linked to hedonic features of a reward, also termed ‘liking’ as opposed to ‘wanting’, which reflects the motivational properties to promote a certain behaviour rather than its hedonic value. However, preclinical studies have shown that stimulation of the µ-opioid (MOP) system in the nucleus accumbens directly enhances incentive motivation (or ‘wanting’) for reward (Pecina and Berridge, 2013). In animals, experimental manipulation of the opioid system can mediate the influence of reward-guided and stimulus-guided decisions on choice (Laurent et al., 2012), increase motivation for different reward types (Mahler and Berridge, 2012) and mediate the motivating influence of cue-triggered reward expectations (Lichtenberg and Wassum, 2017). In humans, evidence for a functional role of the opioid system in mediating ‘wanting’ mainly stems from pharmacological challenges. For instance, MOP agonists and antagonists selectively enhance and decrease processing efficiency in a reward task (Eikemo et al., 2017) and increase and decrease the motivation to view positive valenced stimuli, respectively (Chelnokova et al., 2014). Likewise, opioid receptor antagonists reduced physical effort produced to obtain reward and increased negative facial reactions during reward anticipation (Korb et al., 2019).

In humans, the role of the opioid system in mediating the PIT effect as one further quantification of incentive salience (or ‘wanting’) is less clear. The opioid receptor antagonist naltrexone could decrease alcohol cue-induced activation of the ventral striatum (Myrick et al., 2008) and cue-induced impulsive responding (Mitchell et al., 2007). However, to date, there are only two studies investigating the role of the opioid system in mediating human PIT-like effects (Weber et al., 2016; Wiers et al., 2009), reporting reduced PIT after blockade of the MOP receptor (naltrexone) in healthy humans (Weber et al., 2016) and increased automatic approach tendencies in G+ carriers of the OPRM1 polymorphism to alcohol-associated stimuli (Wiers et al., 2009). The overarching aim of our study was to further elucidate the role of the human opioid system in mediating the PIT effect in both healthy subjects and those with AD.

A common mechanism of quantifying interindividual differences in the human opioid system is the determination of the MOP receptor single nucleotide polymorphism (*OPRM1*). The *OPRM1* gene codes for the MOP receptor, an inhibitory G-protein coupled receptor that binds endogenous opioid peptides such as β-endorphin and enkephalins as well as exogenous opioids such as morphine and heroin (Burns et al., 2019; Kieffer and Gaveriaux-Ruff, 2002). Opioid receptors are distributed widely in the human brain and modulate brain function at all levels of neural integration, including the mesolimbic system as part of the brain’s reward pathway.

Human studies investigating the *OPRM1* polymorphism have suggested a crucial role of this single nucleotide polymorphism (SNP) in AD, treatment response and automatic approach biases to conditioned cues (Chamorro et al., 2012; Filbey et al., 2008; Ray and Hutchison, 2004; Wiers et al., 2009). The A118G (rs1799971) polymorphism of the *OPRM1* gene alters the function of MOP receptors, such that the G variant binds beta-endorphin three times more strongly than the A variant, potentially also affecting receptor availability (Heinz et al., 2005). We henceforth refer to the minor *OPRM1* G-allele carriers as G+ carriers. G+ carriers were shown to report higher subjective alcohol-associated feelings of intoxication (Ray and Hutchison, 2004) and craving (Van Den Wildenberg et al., 2007) and have a higher risk for positive family history (Ray and Hutchison, 2004). However, conflicting results stem from large genome-wide association studies (GWAS) and candidate gene studies (Kong et al., 2017), which could not replicate an association between AD and *OPRM1* genotype, corresponding with a recent report on converging evidence against an association between the *OPRM1* A118G polymorphism and alcohol consumption and sedation (Sloan et al., 2018).

The analyses presented here aimed to answer three questions. (1) Is the *OPRM1* polymorphism associated with the PIT effect across three independent cohorts? (2) Is the association between the PIT effect and the *OPRM1* polymorphism different in patients with AD compared to healthy controls (HCs)? (3) Is the association between the PIT effect and the *OPRM1* polymorphism relevant for treatment outcome in the way that it is different in prospectively relapsing and abstinent patients with AD?

## Methods

### Subjects

All subjects were recruited between 2012 and 2018 as part of a larger study (LeAD study, ClinicalTrials.gov identifiers: NCT01744834, NCT01679145 and NCT02615977) investigating behavioural, genetic and neuroimaging alterations associated with reward-based learning as (a) predictors for the development of AD in a sample of young 18-year-old male subjects recruited from the national registry and (b) the maintenance of AD with respect to relapse and drinking behaviour in a sample of patients suffering from AD and an age, education and sex-matched HC sample (for previously published results of our sample, see, amongst others, Garbusow et al., 2014, 2016, 2019). Thus, this study comprised two independent HC samples that significantly differed with regards to several sociodemographic variables (see Supplemental Table S2 for between-group differences). As previous analyses (Sebold et al., 2016) indicated substantial differences in PIT effects between these cohorts, we did not merge both control samples but instead analysed the influence of the *OPRM1* polymorphism on the PIT effect separately in these two control cohorts (analysis 1).

The assessed samples were a subsample of the three cohorts mentioned above for which genetic data were available: 18-year-old male subjects (*N* = 161, henceforth referred to as young controls), recently detoxified patients with AD (*N* = 186) and age-matched HCs (*N* = 105, henceforth referred to as middle-aged controls). For a precise overview of the selection procedures, see Supplemental Information 1 and Supplemental Figure S1.

For a complete description of exclusion criteria, see Garbusow et al. (2016). Briefly, all subjects were free from psychotropic medication, had no history of substance dependence (DSM-IV, except from AD in the AD group) or current substance use (DSM-IV, except for nicotine use), no other current DSM-IV axis 1 psychiatric or neurological disorders and no borderline personality disorder as assessed by the computer-based Composite International Diagnostic Interview (Jacobi et al., 2013; Wittchen, 1997). Participants’ demographic and clinical characteristics are outlined in Table 1. Participants gave written informed consent before study inclusion. The study was approved by the local ethics committees of the Technical University of Dresden and Charité Universitätsmedizin Berlin.

**Table 1.** Demographic, clinical and neuropsychological characteristics for all cohorts; young controls, middle-aged controls and patients with AD, split by *OPRM1* polymorphism.

Cohort	Alcohol-dependent patients (*N* = 186)			Middle-aged controls (*N* = 105)			Young controls (*N* = 161)		
*OPRM1*	G− (*N* = 154)	G+ (*N* = 32)	Test statistics	G− (*N* = 79)	G+ (*N* = 26)	Test statistics	G− (*N* = 120)	G+ (*N* = 41)	Test statistics
	*M* (*SD*)	*M* (*SD*)		*M* (*SD*)	*M* (*SD*)		*M* (*SD*)	*M* (*SD*)	
*Demographic variables*									
Age	46.17 (10.49)	47.09 (11.03)	*t* = −0.42, *p* = 0.67	43.64 (11.1)	46.04 (10.5)	*t* = −0.99, *p* = 0.33	18.36 (0.2)	18.37 (0.2)	*t* = −0.33, *p* = 0.74
Sex (% male)	84%	81%	χ^2^ = 0.2, *p* = 0.67	83%	81%	χ^2^ = 0.11, *p* = 0.75	100%	100%	NA
Years of education	14.97 (4.07)	14.29 (2.52)	*t* = 1.3, *p* = 0.22	15.98 (3.22)	15.37 (3.32)	*t* = 0.79, *p* = 0.43	11.7 (0.75)	11.51 (1.34)	*t* = 0.85, *p* = 0.4
*Clinical characteristics*									
Anxiety^a^	4.37 (3.41)	4.8 (3.37)	*t* = −0.63, *p* = 0.53	2.32 (2.04)	1.88 (2.21)	*t* = 0.89, *p* = 0.38	2.31 (2.19)	2.92 (2.89)	*t* = −1.2, *p* = 0.23
Depression^b^	3.5 (3.7)	4.33 (3.33)	*t* = −1.23, *p* = 0.23	1.48 (1.98)	1.85 (2.62)	*t* = −0.65, *p* = 0.52	1.67 (1.75)	1.8 (2)	*t* = −0.39, *p* = 0.7
Craving^c^	12.76 (7.94)	12.52 (8.57)	*t* = 0.14, *p* = 0.88	2.4 (2.41)	3.68 (4.03)	*t* = −1.31, *p* = 0.2	3.47 (3.01)	4.65 (3.48)	*t* = −1.91, *p* = 0.7
Impulsivity^d^	31.63 (6.67)	31.84 (5.57)	*t* = −0.19, *p* = 0.85	29.32 (5.4)	28.84 (5.3)	*t* = 0.39, *p* = 0.69	29.99 (5.15)	31.82 (4.56)	*t* = −2.13, ***p*** **=** **0.04**
*Neuropsychological testing*									
Cognitive speed^e^	9.27 (2.76)	9.48 (2.78)	*t* = −0.39, *p* = 0.7	10.58 (2.82)	10.92 (3.78)	*t* = −0.42, *p* = 0.68	11.5 (2.2)	11 (2.59)	*t* = 1.11, *p* = 0.27
Working memory^f^	6.5 (1.93)	6.77 (1.61)	*t* = −0.82, *p* = 0.41	7.41 (1.95)	7.62 (2.43)	*t* = −0.40, *p* = 0.69	8.04 (1.95)	8.02 (2.21)	*t* = 0.04, *p* = 0.96

The variables were assessed by means of ^a^the anxiety and ^b^depression subscale of the Hospital Depression and Anxiety Questionnaire; ^c^the Obsessive Compulsive Drinking Scale, ^d^the Barratt Impulsiveness Scale and the following subtest of the Wechsler Intelligence Test: ^e^the Digit Symbol Substitution Test and ^f^the Digit Span Backwards Test.

AD: alcohol dependence.

To define relapse across patients with AD, a three-month follow-up was performed using the Time Line Follow Back procedure (Sobell and Sobell, 1992). Relapse was defined as at least five standard drinks (e.g. one standard drink = 0.33 L beer) on one occasion for male participants and at least four standard drinks for female participants according to the World Health Organization (WHO; 2014) definition of high-risk consumption. A total of 51 patients were classified as relapsers (of whom 37 were G− and 14 were G+ carriers), whereas 94 patients were classified as abstainers (of whom 78 were G− and 16 were G+ carriers). The remaining 41 patients could not be contacted during the follow-up period.

### Task

**Figure 1.**
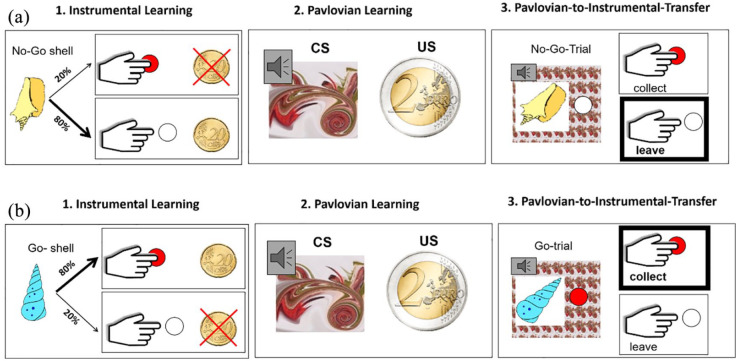
Phases 1–3 of the paradigm for (a) the ‘No-Go’ trial and (b) the ‘Go’ trial. 1. Instrumental learning: The subject’s task was to move a dot towards the stimulus by repeated button presses in order to collect it or to do nothing within two seconds. These two instrumental choices resulted in monetary wins or losses, presented immediately after each trial via a picture of a 20€ coin for 1.5 seconds. Feedback was probabilistic. A ‘Go’ shell was rewarded in 80% and punished in 20% of trials if collected and vice versa if not collected. A ‘No-Go’ shell was rewarded in 80% and punished in 20% of the trials if not collected and vice versa if collected. 2. Pavlovian learning: Neutral fractal and audio stimulus compounds (CS) are repeatedly paired with monetary outcomes (US: e.g. here a 2€ coin). 3. Pavlovian-to-instrumental transfer (PIT) phase: Subjects performed the instrumental task in nominal extinction, that is, no explicit monetary outcomes were presented (A. leave button to not collect a ‘No-Go’ shell and B. press button to collect ‘Go’ shell superimposed on the audiovisual Pavlovian stimulus; here: the Pavlovian stimulus previously paired with 2€ and the respective tone pitch).

We used a PIT task as previously described (Garbusow et al., 2014, 2016; Sommer et al., 2017). The task consisted of four phases (of which the first three phases are depicted in Figure 1): (a) instrumental learning, (b) Pavlovian learning, (c) PIT and (d) forced choice task followed by a rating scale of the stimuli.

#### Instrumental learning

Subjects had to learn to collect ‘Go’ shells and leave ‘No-Go’ shells by repeatedly pressing a button while receiving probabilistic feedback. In order to collect a shell, subjects had to move a red dot onto the selected shell by repeated button presses within two seconds. We instructed the subjects to maximise their profit. For this, they should use the probabilistic feedback to find out via trial and error what is a ‘good shell’, which in ‘most cases’ lead to wins when collected, and leave ‘bad shells’, which in ‘most cases’ lead to wins when not collected. Each button press moved the red dot a fraction of the way towards the shell. To collect a ‘Go’ shell correctly, subjects had to press the button five or more times, and to leave a ‘No-Go’ shell, subjects had to perform between zero and four button presses. The subjects did not know about the number of button presses, but we instructed them to press the button as often as possible to collect a shell to maximise instrumental performance. Correct responses were rewarded with 20 cents in 80% of trials and punished with a loss of 20 cents in 20% of trials, and for wrong responses it was vice versa (see Figure 1.1 for ‘Go’ and ‘No-Go’ trials). The shell set consisted of six different shells (three ‘Go’ shells and three ‘No-Go’ shells).

Participants performed 60–120 trials, depending on their performance. In order to ensure that all subjects were at comparable performance levels before advancing to the PIT part, a learning criterion was enforced (80% correct choices over 16 trials after a minimum of 60 trials).

#### Pavlovian learning

Pavlovian learning consisted of 80 trials in which compound visual and auditory stimuli (CS) were predictive of distinct monetary rewards or punishments (unconditioned stimulus (US); Figure 1.2). Each trial began with a three-second presentation of a compound CS (fractal picture and tone) which was then followed by a three-second presentation of two fixation crosses (on the left and right side of the screen). Then, the US (monetary reward or punishment) was presented for threeseconds on the side where the CS had not been presented. Subjects were instructed to view the CS–US pairings passively and to memorise these associations. The set of CS consisted of six stimuli of which each was paired with positive (+2€/+1€), neutral (0€) or negative (−1€/−2€) outcomes, henceforth referred to as ‘money CS’.

#### PIT phase

Subjects performed 162 trials of the instrumental task again, this time without outcome feedback. Subjects were instructed that their choices still counted towards the final monetary outcome (so-called nominal extinction). The instrumental stimuli superimposed one of the money CSs presented during Pavlovian training (Figure 1.3), or one of four beverage stimuli (results not presented here, but see (Schad et al., 2019a; Sekutowicz et al., 2019; Sommer et al., 2017, 2020). Each instrumental stimulus (three ‘Go’ shells and three ‘No-Go’ shells) was combined with each money CS (fractal stimulus previously associated with either of −2€, −1€, 0€, +1€, +2€) for three times, resulting in 90 trials, which were of primary interest for this study. Each trial lasted 3.6 seconds.

#### Forced-choice task

This part of the task aimed to verify the acquisition of Pavlovian learning. In each trial, subjects had to choose between two sequentially presented compound money CSs from the Pavlovian training, each presented for two seconds. All possible compound CS pairings were presented three times in an interleaved randomised order.

#### Pleasantness ratings

After the task, subjects were asked to rate the pleasantness of the CSs (fractals and shells) from the Pavlovian learning phase and the instrumental learning phase on a Likert scale from 1 to 7 on the screen.

### Genotyping

To genotype our sample, DNA was extracted semi-automatically with a Chemagen Magnetic Separation Module (PerkinElmer, Waltham, MA) from whole blood. All samples were genotyped with the Illumina Infinium Psych Array Bead Chip (Illumina, San Diego, CA). We assessed rs1799971, a SNP that is an A-to-G substitution (A118G), resulting in a functional amino acid substitution (Asn40Asp; Hartwell et al., 2020).

Because of the limited sample size, G-allele carriers (AG and GG) were grouped together. This approach is in keeping with precedent in the field (Persson et al., 2019; Way et al., 2009).

### Behavioural analyses

Data were analysed using the R programming language (R Foundation for Statistical Computing, Vienna, Austria). Demographic, clinical and neuropsychological comparisons between G+ and G− *OPRM1* carriers were examined using chi-square and *t*-tests (Table 1).

Analysis of the PIT phase was of primary interest, but we analysed all other phases as well (see Supplemental Information 6, Supplemental Information 7, Supplemental Information 8 and Supplemental Information 10). In the PIT phase, the PIT effect reflects the interaction between the valence of the background stimulus and the accuracy of the foreground instrumental action. We were specifically interested whether the *OPRM1* genotype covaried with PIT effect, that is, the way that positive and negative stimuli influence ‘Go’ and ‘No-Go’ actions. More precisely, we asked whether the genetic phenotype would interact with the extent to which a positive stimulus facilitates ‘Go’ responses but impairs ‘No-Go’ responses and, vice versa, a negative stimulus facilitates ‘No-Go’ responses but impairs ‘Go’ responses.

As outlined in the introduction, the analyses presented here aimed to elucidate: (a) the association between the *OPRM1* polymorphism and the PIT effect, (b) the clinical relevance of this association for AD and (c) the relevance of this association for treatment outcome. Across these different analyses, we coded a participant’s accuracy of the PIT phase as correct (1) if a ‘Go’ shell was collected or a ‘No-Go’ shell was left, and as false (0) if a ‘No-Go’ shell was collected or a ‘Go’ shell was left. We used a binomial mixed effect regression as implemented in the lme4 package (Bates et al., 2015). We regressed the participant’s accuracy (correct or incorrect) on Pavlovian valence (negative, neutral or positive, dummy coded with neutral as the reference), instrumental action (‘Go’ or ‘No-Go’, coded as 0.5 and −0.5) and *OPRM1* polymorphism (G− or G+, coded as −0.5 and +0.5) and tested for interaction between these factors. Subjects were added as random effects (random intercept model). We performed model comparisons to ensure that this model was the best-fitting model across subjects (see Supplmenetal Information 2).

#### Analysis 1: Association between the PIT effect and the *OPRM1* polymorphism across cohorts

To test whether the *OPRM1* polymorphism was associated with the PIT effect in all three cohorts, we performed the above-described analysis for all three cohorts separately (Supplemental Figure S1).

#### Analysis 2: Alcohol-related group differences for the association between the PIT effect and the *OPRM1* polymorphism

To test whether the interaction between the PIT effect and the *OPRM1* polymorphism was significantly different between HCs and patients with AD, we performed the above-described regression model (see analysis 1) but additionally added group (HC or AD, coded as 0.5 and −0.5) as an additional fixed effect and allowed interaction with all predictors (Supplemental Figure S1). For this analysis, we only included patients with AD and middle-aged control subjects (who were initially sampled as a comparison group of patients with AD). Both groups profoundly differed across several socio-economic and clinical variables (Supplemental Table S2). Increased depression, anxiety, craving and impulsivity as well as reduced cognitive speed and working memory are features instead of confounders of AD. Thus, as suggested by Miller and Chapman (2001), we did not control for these variables. Years of education was the only variable we added as a covariate because groups significantly differed in these variables despite our efforts of matching.

#### Analysis 3: Relapse-related group differences for the association between the PIT effect and the *OPRM1* polymorphism

To test whether the interaction between the *OPRM1* polymorphism and the PIT effect was significantly different between patients with AD who relapsed and those who remained abstinent, we performed the above described regression analysis (see analysis 1) but added relapse (relapsers or abstainers, coded as 0.5 and −0.5) as an additional fixed factor and allowed interaction with all predictors. For this analysis, we only included patients with AD for whom relapse data were available (*n* = 145; Supplemental Figure S1). Relapsing patients did not differ from abstaining patients in any demographic or clinical variables, except for craving (where relapsing patients had significantly higher OCDS scores (Anton et al., 1995; Mann and Ackermann, 2000) than abstaining patients (*t* = −2.66, *p* = 0.01). Thus, we added craving as a covariate of no interest in this analysis.

### Post hoc analyses

For analyses 2 and 3, we were particularly interested in how the PIT effect was modulated by the *OPRM1* polymorphism and whether this depended on group, respectively. Thus, in our post hoc analyses, we focused on these contrasts (analysis 2: G+ vs. G− carriers/HCs vs. ADs; analysis 3: G+ vs. G− carriers/relapsers vs. abstainers) and considered effects as significant when they survived Bonferroni correction for four comparisons (*p* < 0.01).

## Results

### Genotyping

Genotyping resulted in 353 participants homozygous for the major A allele, 89 participants with the AG combination and 10 participants homozygous for the G allele. *OPRM1* genotype distribution did not significantly differ from Hardy–Weinberg equilibrium (χ^2^_(*df* = 1)_ = 2.31, *p* = 0.13).

Demographic, clinical and neuropsychological comparisons between G+ and G− carriers in all three cohorts indicated no group differences (Table 1), except from increased self-reports of impulsivity assessed via BIS-15 (Meule, 2011) in G+ carriers compared to G− carriers in young healthy adults. Moreover, we found no evidence for a functional association between the *OPRM1* polymorphism and AD. Descriptively, there were proportionally more G+ carriers among the HCs compared to the AD group – from the literature we would have expected the reverse results – although this difference was formally not statistical significant (χ^2^_(*df* = 1)_ = 3.62, *p* = 0.06). Also, we found no evidence for a functional association between the *OPRM1* polymorphism and relapse (χ^2^_(*df* = 1)_ = 1.60, *p* = 0.21).

### Behavioural data

#### Analysis 1: Association between the PIT effect and the *OPRM1* polymorphism across cohorts

The first aim of this study was to test whether the *OPRM1* polymorphism influences the PIT effect across three independent cohorts. In all three cohorts we found a significant PIT effect, that is, the interaction between Pavlovian valence (negative, neutral or positive) and instrumental action (‘Go’ or ‘No-Go’; Table 2), indicating that positive stimuli facilitated ‘Go’ responses but impaired ‘No-Go’ responses, whereas negative stimuli facilitated ‘No-Go’ responses but impaired ‘Go’ responses.

**Table 2.** Results of analysis 1. Effects of the regression analysis from the PIT part for all three cohorts.

Group	Alcohol-dependent patients (*N* = 186)		Middle-aged controls (*N* = 105)		Young controls (*N* = 161)	
	χ^2^	*p*-Value	χ^2^	*p*-Value	χ^2^	*p*-Value
Pavlovian CS valence	11.723	**0.003**	5.599	0.061	15.105	**0.001**
Instrumental behavior	7.057	**0.008**	13.108	**0.0003**	0.159	0.690
*OPRM1* polymorphism	0.002	0.963	0.046	0.831	0	0.994
Pavlovian valence × instrumental behavior	2074.63	**<0.0001**	912.67	**<0.0001**	365.68	**<0.0001**
Pavlovian valence × *OPRM1* polymorphism	0.224	0.894	0.074	0.964	0.629	0.730
Instrumental behavior × *OPRM1* polymorphism	13.917	**0.0002**	18.930	**<0.0001**	7.757	**0.005**
Pavlovian valence × instrumental behavior × *OPRM1* polymorphism	12.723	**0.002**	9.027	**0.011**	20.691	**<0.0001**

All interaction effects with the *OPRM1* polymorphism in the young control cohort remained significant after controlling for self-reports of impulsivity, which was significantly different between G+ and G− carriers in this cohort (see Table 1). Statistically significant values are shown in bold.

PIT: Pavlovian-to-instrumental transfer; CS: conditioned stimulus.

**Figure 2.**
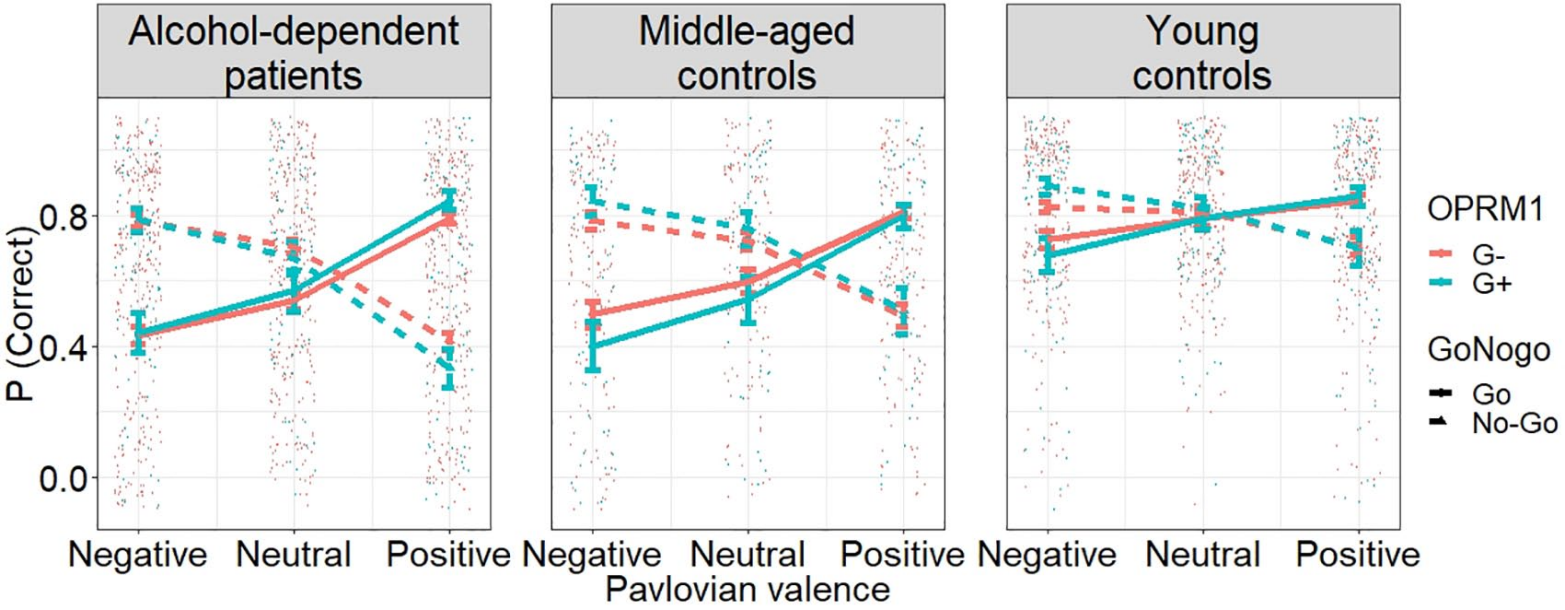
Results of the PIT phase as a function of group (patients with alcohol dependence (AD), middle-aged controls and young controls) and *OPRM1* polymorphism. Each panel shows the PIT effect in the respective group, that is, there was a significant influence of Pavlovian background valence on instrumental action (accuracy: percent correct choices), here visualised by the slope of the lines. Crucially, in each of the three cohorts, this was steeper in G+ carriers compared to G− carriers, as indicated by the three-way interaction between *OPRM1* polymorphism, Pavlovian valence and instrumental action (analysis 1), that is, in each of the three independent cohorts, the PIT effect was modulated by the *OPRM1* polymorphism. However, this was not different between alcohol-dependent patients and matched middle-aged controls (analysis 2).

In all groups, respectively, we found no interaction between Pavlovian valence and *OPRM1* polymorphism. However, the *OPRM1* polymorphism interacted with instrumental action (Table 2). Crucially, we found a three-way interaction between Pavlovian valence, instrumental action and *OPRM1* polymorphism in all cohorts. This result suggests that the *OPRM1* polymorphism strongly interacts with the PIT effect in all three independent cohorts. In fact the PIT effect was significantly higher in G+ carriers compared to G− carriers (Figure 2 and Table 2).

To rule out that our PIT-related *OPRM1* effect was simply due to the fact that G+ carriers showed stronger cue-induced modulation of liking, we further performed analyses of the rating data of the Pavlovian stimuli (pleasantness ratings; Supplemental Information 10). To this end, we first tested whether the *OPRM1* polymorphism was associated with ratings of the stimuli, depending on the Pavlovian valence. In all cohorts, the *OPRM1* polymorphism did not interact with Pavlovian valence (Supplemental Information 10). Moreover, adding the rating data as an additional covariate in our PIT analyses, all interaction between the *OPRM1* polymorphism, Pavlovian valence and instrumental action remained significant (patients with AD: *p* = 0.0004; middle-aged controls: *p* = 0.006; young controls: *p* < 0.0001).

#### Analysis 2: Alcohol-related group differences for the association between the PIT effect and the *OPRM1* polymorphism

The second aim of this study was to test whether the interaction between the PIT effect and *OPRM1* polymorphism was significantly different between patients with AD and HCs. This analysis indicated a three-way interaction between Pavlovian valence, instrumental action and group and also a three-way interaction between Pavlovian valence, instrumental action and *OPRM1* polymorphism. Thus, AD and the *OPRM1* polymorphism were significantly and independently associated with the strength of the PIT effect per se (see Figure 2). Moreover, we found a three-way interaction between instrumental action, group and *OPRM1* polymorphism. However, the four-way interaction between Pavlovian valence, instrumental action, group and *OPRM1* polymorphism was not statistically significant (Table 3). Thus, the interaction between the PIT effect and the *OPRM1* polymorphism was not statistically different between patients with AD and matched control subjects (Figure 2).

**Table 3.** Results of analysis 2. Effects of the regression analysis from the PIT part where we tested whether the interaction between the PIT effect and the *OPRM1* polymorphism was significantly different between patients with AD and HCs.

	χ^2^	*p*-Value
Pavlovian valence	13.183	0.001
Instrumental action	18.391	**<0.0001**
*OPRM1* polymorphism	0.007	0.933
Group	2.316	0.128
Years of education	7.651	0.006
Pavlovian valence × instrumental action	2888.726	**<0.0001**
Pavlovian valence × *OPRM1* polymorphism	0.031	0.984
Instrumental action × *OPRM1* polymorphism	0.374	0.540
Pavlovian valence × group	3.661	0.160
Instrumental action × group	4.187	0.041
*OPRM1* polymorphism × group	0.015	0.901
Pavlovian valence × instrumental action × *OPRM1* polymorphism	16.909	**<0.0001**
Pavlovian valence × instrumental action × group	22.695	**<0.0001**
Pavlovian valence × *OPRM1* polymorphism × group	0.257	0.880
Instrumental action × *OPRM1* polymorphism × group	30.727	**<0.0001**
Pavlovian valence × instrumental action × *OPRM1* polymorphism × group	0.318	0.853

HC: healthy control.

#### Analysis 3: Relapse-related group differences for the association between the PIT effect and the *OPRM1* polymorphism

**Figure 3.**
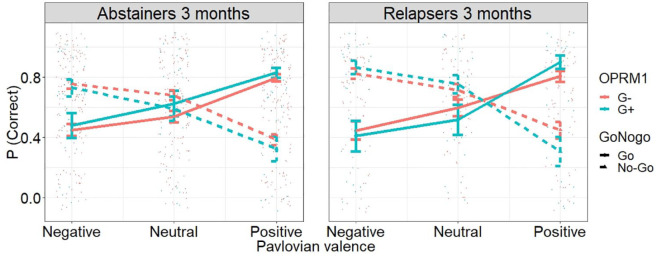
Results of the PIT phase as a function of treatment outcome (abstainers vs. relapsers) and *OPRM1* polymorphism (analysis 3). Patients with AD who relapsed showed a stronger interaction between the PIT effect and the *OPRM1* polymorphism compared to patients with AD who remained abstinent. Moreover, G+ carriers showed a strong and significant interaction between the PIT effect and treatment outcome, whereas G− carriers did not.

Last, we tested whether the observed interaction between the *OPRM1* polymorphism and the PIT effect was associated with relapse. Again, we found a three-way interaction between the *OPRM1* polymorphism, Pavlovian valence and instrumental action (Table 4). In addition, we observed an interaction between relapse status and instrumental action, and a three-way interaction between Pavlovian valence, instrumental action and relapse. This interaction was further modulated by the *OPRM1* polymorphism, resulting in the expected four-way interaction between Pavlovian valence, instrumental action, *OPRM1* polymorphism and relapse status (Figure 3 and Table 4). Thus, the interaction between the *OPRM1* polymorphism and the PIT effect was statistically different between patients with AD who prospectively relapsed and those who remained abstinent. Post hoc tests indicated that the interaction between Pavlovian valence, instrumental action and the *OPRM1* polymorphism was only significant for relapsers (*p* < 0.0001) but not for abstainers (*p* = 0.328). Moreover, the interaction between Pavlovian valence, instrumental action and relapse was significant for G+ carriers (*p* < 0.0001) but not for G− carriers (*p* = 0.09).

**Table 4.** Results of analysis 3. Effects of the regression analysis from the PIT part where we tested whether the interaction between the PIT effect and the *OPRM1* polymorphism was significantly different between relapsers and abstainers.

	χ^2^	*p*-Value
Pavlovian valence	10.27	**0.006**
Instrumental action	0.002	0.965
*OPRM1* polymorphism	0.324	0.569
Relapse	0.706	0.401
Craving	0.053	0.817
Pavlovian valence × instrumental action	1535.13	**<0.0001**
Pavlovian valence × *OPRM1* polymorphism	0.426	0.808
Instrumental action × *OPRM1* polymorphism	11.706	**0.001**
Pavlovian valence × relapse	0.513	0.774
Instrumental action × relapse	12.786	**<0.0001**
*OPRM1* polymorphism × relapse	0.042	0.838
Pavlovian valence × instrumental action × *OPRM1* polymorphism	16.786	**0.001**
Pavlovian valence × instrumental action × relapse	13.647	**0.001**
Pavlovian valence × *OPRM1* polymorphism × relapse	0.571	0.752
Instrumental action × *OPRM1* polymorphism × relapse	1.988	0.159
Pavlovian valence × instrumental action × *OPRM1* polymorphism × relapse	30.347	**<0.0001**

## Discussion

To explore and further understand the behavioural and genetic underpinnings of ‘wanting’ as an expression of incentive salience attribution in humans and to bridge the gap to preclinical results, we investigated the association between the *OPRM1* polymorphism, PIT effect and relapse across a large cohort of patients with AD and two independent cohorts of HCs.

We demonstrate that (a) in all three independent cohorts, G+ carriers showed an increased PIT effect; (b) there is no difference between patients with AD and HCs in the interaction between *OPRM1* and PIT; but (c) when merely investigating AD, relapsing patients carrying the G+ allele showed an increased PIT effect as opposed to abstaining patients, who did not show an association between *OPRM1* genotype and PIT. We henceforth discuss these three main results.

### Analysis 1: Association between the PIT effect and the *OPRM1* polymorphism across cohorts

The first analysis demonstrated a clear association between the *OPRM1* genotype and PIT in three independent human cohorts. Two studies have previously investigated the role of the human opioid system in PIT-like effects in healthy human subjects. By using a pharmacological challenge, Weber et al. (2016) demonstrated that naltrexone reduces PIT effects for primary reinforcers (e.g. food rewards). We here demonstrate that the opioid system is also involved in modulating PIT effects for secondary reinforcers (e.g. monetary rewards). Beyond this, the experimental design from Weber et al. (2016) also differed in several other aspects from our study. Weber et al. (2016) focused on the positive ‘limb’ of the PIT effect (the extent to which positive stimuli affect responses), whereas our paradigm also enabled us to examine the negative ‘limb’ of the PIT effect (the extent to which negative stimuli affect responses). Moreover, our instrumental task included both ‘Go’ and ‘No-Go’ responses, whereas the instrumental task by Weber et al. (2016) merely included a ‘Go’ component. Thus, in line with previous investigations (Guitart-Masip et al., 2011, 2014; Swart et al., 2017), our experimental manipulation enabled us to test for more complex valence–action interactions. These previous tasks in line with our results have identified a potentially phylogenetically induced bias for congruent action–valence responses (e.g. better performance when a ‘Go’ response was acquired to win) compared to incongruent action valence (e.g. when a ‘No-Go’ response was acquired to win).

A second study published by Wiers et al. (2009) investigated automatic appetitive action tendencies in male heavy-drinking carriers of the *OPRM1* G allele. Heavy-drinking G+ carriers showed increased automatic approach tendencies not only to alcohol-associated stimuli but also to other appetitive stimuli (Wiers et al., 2009). This is in line with our finding of increased behavioural modulation in the presence of appetitive cues in AD G+ carriers. However, Wiers et al. did not include a control group in their study design and only included male sex, which limits generalisability and comparability to our results.

In summary, our data support the notion that the *OPRM1* polymorphism serves as one biological agent associated with human PIT effect in both AD patients and HCs.

### Analysis 2: Alcohol-related group differences for the association between the PIT effect and the *OPRM1* polymorphism

We did not find a significantly different association between the PIT effect and the *OPRM1* polymorphism between patients with AD and HCs, which partly reflects the ongoing debate and contradictory results published so far on the association between the *OPRM1* genotype and AD (Hendershot et al., 2016; Kong et al., 2017; Ray and Hutchison, 2004; Sloan et al., 2018). Instead, we found that AD and the *OPRM1* polymorphism are independent factors that both increase the PIT effect. Moreover, we found an interaction between instrumental action, *OPRM1* polymorphism and group, indicating that the opioid system differently affects instrumental responses in AD patients and HCs. Exploratory post hoc analyses (Supplementary Information 4) indicated that AD G+ carriers showed increased ‘Go’ responses compared to ‘No-Go’ responses, whereas HC G+ carriers showed increased ‘No-Go’ responses compared to ‘Go’ responses. Of note, a positive PIT effect is accompanied by an overall increase of ‘Go’ responses, while a negative PIT effect is accompanied by an overall increase in ‘No-Go’ responding. Thus, the *OPRM1* polymorphism may influence the positive PIT effect in patients with AD and the negative PIT effect in HC. A core feature of AD is the persistent substance consumption despite the negative consequences of consumption (Stacy and Wiers, 2010). We speculate that this paradox might partly be explained by an increased responsiveness of patients with AD to positively conditioned cues, which is stronger in G+ carriers. On the other hand, an increased responsiveness to negative stimuli might reveal a protective mechanism of healthy G+ carriers (S3 and S4). Clearly, future studies need to validate this speculation.

### Analysis 3: Relapse-related group differences for the association between the PIT effect and the *OPRM1* polymorphism

Only relapsers but not abstainers showed a significant interaction between the PIT effect and the *OPRM1* polymorphism. Moreover, only relapsing G+ carriers showed an increased PIT effect compared to abstainers, whereas there was no difference between the PIT effect in relapsers and abstainers in G− carriers. One speculative interpretation of these findings is that there may be two pathways to relapse, and that these fundamentally differ with regard to the *OPRM1* polymorphism and the PIT effect. On the one hand, in G+ carriers, the mechanisms driving PIT might also be related to relapse, whereas in G− carriers, these mechanisms could be less related to relapse. Our finding of an increased PIT effect in relapsing AD G+ carriers might also be relevant for precision medicine, particularly in the light of the ongoing discussion of the *OPRM1* polymorphism as a potential biomarker for the effectiveness of naltrexone treatment (Chamorro et al., 2012; Hartwell et al., 2020; Oslin et al., 2003; Setiawan et al., 2012; Ziauddeen et al., 2016). Strikingly, treatment response to naltrexone was also particularly high in patients with AD classified as reward drinkers (Mann et al., 2018; Witkiewitz et al., 2019) and reduced craving, most notably in social drinkers, who had high positive alcohol expectancies (Palfai et al., 1999).

Similar considerations might be relevant to nalmefene, the MOP antagonist and partial κ-agonist, recently approved for the treatment of AD (Gual et al., 2013), with similarly conflicting results. According to a meta-analysis, the drug is able to improve behavioural outcomes in patients with AD (Mann et al., 2016), while others show that it has a limited efficacy in AD therapy (Palpacuer et al., 2015; Soyka and Muller, 2017). Nalmefene administered in a modified ‘Go’/‘No-Go’ paradigm mildly reduced vigor to alcoholic cues in patients with AD (Gal et al., 2019). However, no major differences were observed between the treatment group and the placebo group with respect to behavioural and neural correlates of approach/avoidance tendencies. Given our data, future studies could investigate whether naltrexone and/or nalmefeme might be particularly effective in alcohol-dependent patients who are G+ carriers and additionally show large PIT effects.

### Outlook: How does *OPRM1* influence neural reward processing?

The neural correlates of PIT have been associated with relapse in AD within the mesolimbic reward system (Garbusow et al., 2016; Sekutowicz et al., 2019; Sommer et al., 2020) and could predict future drinking behaviour in adolescents (Sekutowicz et al., 2019). Recent studies have suggested a direct link between the *OPRM1* polymorphism and the mesolimbic dopaminergic system. For instance, by using a mouse model of the *OPRM1* A118G SNP, Popova et al. (2019) demonstrated that A- and G-allele carriers show significantly different regulation of mesolimbic dopaminergic firing. One potential underlying mechanism is that MOP receptors (which are affected by the *OPRM1* polymorphism) mediate opioid-induced disinhibition of midbrain dopaminergic neurons (Jalabert et al., 2011; Jhou et al., 2012; Matsui et al., 2014). Recent work in rodents has proven that optogenetic manipulations of those dopaminergic neurons can bidirectionally modulate online action selection (Howard et al., 2017). Thus, we speculate that the *OPRM1* polymorphism is associated with the extent to which Pavlovian stimuli functionally activate the mesolimbic dopaminergic system in AD. This speculation is in line with functional magnetic resonance imaging studies using cue reactivity paradigms in substance-dependent individuals. For instance, some studies suggest that AD G+ carriers display increased neural responses to alcohol-associated stimuli in mesocorticolimbic areas (Bach et al., 2015; Courtney et al., 2015; Filbey et al., 2008; but see Schacht et al., 2013). In line with this, humanised mice carrying the G+ allele of the *OPRM1* polymorphism displayed increased striatal dopamine release in response to an intravenously infused alcohol dose (Ramchandani et al., 2011). Clearly, future studies should further investigate how the *OPRM1* polymorphism affects the underlying neural mechanisms of the PIT effect in humans.

### Limitations

The generalisability of our results is limited by the lack of preregistration, additional analyses designed after study protocol and the use of single gene analyses. The correlational nature of the analyses only allows speculation about causal relationships and needs to be further validated in a longitudinal design. Even though candidate genes as opposed to large-scale GWAS studies have come into disrepute, we believe that there is still a high relevance in connecting single genes and their respective pathways to specific neurocognitive processes and thus providing the opportunity for more specific interventions in precision medicine (Deb et al., 2010; Di Martino et al., 2020). Another limitation of our design is that the procedure used here to indicate Pavlovian learning (task phase 4) was not designed to detect between-group effects but instead served to identify subjects who did not learn the Pavlovian contingencies (Supplemental Information 8). Across all cohorts, subjects could almost perfectly identify the best Pavlovian stimuli, and these ceiling effects potentially lowered statistical power to detect differences in Pavlovian learning. Several studies across humans and animals have demonstrated that individuals who attribute incentive salience to reward predicting stimuli through Pavlovian conditioning (so called sign-trackers) will also show an increased PIT effect (Garofalo and di Pellegrino, 2015; Schad et al., 2019b). Future studies should therefore use more sensitive methods to identify sign-tracking humans (such as eye-tracking; Schad et al., 2019b) and test the role of the *OPRM1* polymorphism in this phenomenon. One further limitation is the relatively small sample size of relapsers versus abstainers in analysis 3. Importantly, the group of G+ carriers that relapsed versus abstained was 16 versus 14, respectively. Thus, future stratification studies need to replicate our findings in larger sampling sizes, for example by oversampling G+ carriers in AD.

### Summary

This study presents strong evidence for an association between the *OPRM1* polymorphism and the PIT effect in both patients with AD and HCs. It is the first to show that the *OPRM1* polymorphism modulates the extent to which Pavlovian stimuli exert control over behaviour and suggests a functional difference of this gene–behaviour interaction between relapsers and abstainers.

## Supplemental Material

sj-docx-1-jop-10.1177_0269881121991992 – Supplemental material for Association of the OPRM1 A118G polymorphism and Pavlovian-to-instrumental transfer: Clinical relevance for alcohol dependence Click here for additional data file. Supplemental material, sj-docx-1-jop-10.1177_0269881121991992 for Association of the OPRM1 A118G polymorphism and Pavlovian-to-instrumental transfer: Clinical relevance for alcohol dependence by Miriam Sebold, Maria Garbusow, Deniz Cerci, Ke Chen, Christian Sommer, Quentin JM Huys, Stephan Nebe, Michael Rapp, Ilya M Veer, Ulrich S Zimmermann, Michael N Smolka, Henrik Walter, Andreas Heinz and Eva Friedel in Journal of Psychopharmacology
