# Supplementary material for: Association of the OPRM1 A118G polymorphism and Pavlovian-to-instrumental transfer: Clinical relevance for alcohol dependence
Source: J Psychopharmacol. 2021 Mar 16;35(5):566–78. doi: 10.1177/0269881121991992 (PMC8155738; doi:10.1177/0269881121991992)
Supplement: sj-docx-1-jop-10.1177_0269881121991992 – Supplemental material for Association of the OPRM1 A118G polymorphism and Pavlovian-to-instrumental transfer: Clinical relevance for alcohol dependence [file sj-docx-1-jop-10.1177_0269881121991992.docx]

Supplementary Information: The association of the OPRM1 A118G polymorphism and Pavlovian-to-instrumental transfer: clinical relevance for alcohol dependence

Sebold, M.^1,2^, Garbusow, M.^1^, Cerci, D.^3^, Chen K. ^1^, Sommer C.^4^, Huys Q.J.M.^6,7^, Nebe S.^8^, Rapp M.^2^, Veer I.M.^1^, Zimmermann U.S.^4,5^, Smolka M.N.^4^, Walter, H.^1^, Heinz, A.^1,^ Friedel E.^1,9^

^1^ Charité – Universitätsmedizin Berlin, corporate member of Freie Universität Berlin, Humboldt-Universität zu Berlin, and Berlin Institute of Health, Department of Psychiatry and Psychotherapy, 10117 Berlin, Germany

^2^ Department for Social and Preventive Medicine, University of Potsdam, Potsdam, Germany,

^3^ Klinik für Forensische Psychiatrie, Universitätsmedizin Rostock, Rostock, Germany

^4^ Technical University of Dresden, Dresden, Germany

^5^ Department of Addiction Medicine and Psychotherapy, kbo Isar-Amper-Klinikum, Munich

^6^ Division of Psychiatry, University College London, London, United Kingdom

^7^ Max Planck UCL Centre for Computational Psychiatry and Ageing Research, University College London, London, United Kingdom

^8^ Department of Economics, University of Zurich, Zurich, Switzerland

^9^ Berlin Institute of Health (BIH), 10178 Berlin, Germany

# SI 1: Sample selection

Originally, we had behavioral data of 542 subjects (221 alcohol-dependent (AD) patients, 129 middle-aged controls, 192 young controls). However, due to missing data in the genetic information (27 AD patients, 4 middle-aged controls, 19 young controls) or due to insufficient performance in the forced choice task (indicating low Pavlovian learning: 8 AD patients, 17 middle aged controls, 12 young controls), we had to exclude several subjects (Figure S1), resulting in the final data sets reported throughout the manuscript for the respective analsysis.


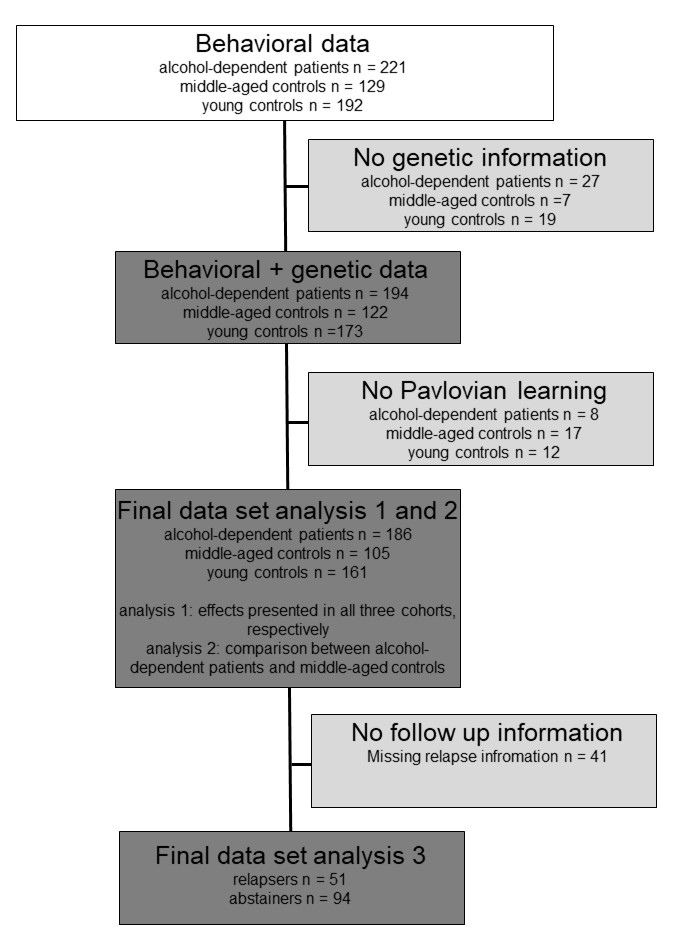


Figure S1: Sample selection procedure.

# SI 2: Model Comparisons

We performed several generalized linear mixed models (across all subjects) and compared model fits. The first model (henceforth referred to *“1. linear-accuracy”*) included Pavlovian valence as a linear contrast (-2,-1,0,1,2), instrumental action (Go, No-Go, coded as 0.5 and -0.5) and OPRM1 polymorphism (G-, G+, coded as -0.5 and +0.5) on the participant’s accuracy (Correct, Incorrect). This model (without the OPRM1 polymorphism) has been used to model performance of the PIT paradigm in a recent sample of the LeAD study (Sommer et al., 2017). The next model (henceforth referred to as “*2. non-linear-accuracy”*) included Pavlovian valence as non-linear contrast (Negative, Neutral, Positive, dummy coded with neutral as reference), instrumental action (Go, No-Go, coded as 0.5 and -0.5) and OPRM1 polymorphism (G-, G+, coded as -0.5 and +0.5) on the participant’s accuracy (Correct, Incorrect). We additionally fitted reduced versions of these two models: a linear accuracy model without the OPRM1 polymorphism, henceforth referred as *“1a: linear accuracy no OPRM1”*, a linear accuracy model without the instrumental action: henceforth referred as *“1b: linear accuracy no Instrumental action”* and the same for the non-linear accuracy model: a non-linear accuracy model without the OPRM1 polymorphism, henceforth referred as *“2a: non-linear accuracy no OPRM1”*, a non-linear accuracy model without the instrumental action: henceforth referred as *“2b: non-linear accuracy no Instrumental action”.* Last, we fitted an accuracy model without Pavlovian valence, henceforth referred as *“3: accuracy no Pavlovian valence”*

We used the *anova* function of the *stats* package to perform model comparisons. This analysis indicated that the non-linear accuracy model was the best fitting model (lowest AIC score and highest Log Likelihood, Table S1).

Table S1: Results of the model comparisons

| **Model** | **AIC** | **Log Likelihood** |
| --- | --- | --- |
| 1. linear-accuracy | 42164 | -21073 |
| 1a: linear accuracy no OPRM1 | 42182 | -21086 |
| 1b: linear accuracy no Instrumental action | 45479 | -22735 |
| **2. non-linear-accuracy** | **41851** | **-20911** |
| 2a: non-linear accuracy no OPRM1 | 41900 | -20943 |
| 2b: non-linear accuracy no Instrumental action | 45471 | -22728 |
| 3: accuracy no Pavlovian valence | 45470 | -22730 |

# SI 3: Group comparisons for demographic and clinical characteristics

Table S2: OPRM1 independent group comparisons for alcohol dependent patients, middle aged controls and young healthy controls

| **Cohort** | **Alcohol-dependent patients** | **Middle-aged controls** | **Young controls** | **Test Statistics** | | | |
| --- | --- | --- | --- | --- | --- | --- | --- |
|  | **mean (sd)** | **mean (sd)** | **mean (sd)** | **Anova statistics** | **Alcohol-dependent patients vs. Middle-aged controls** | **Alcohol-dependent patients vs. Young controls** | **Middle-aged controls vs. Young controls** |
| **Demographic Variables** | |  |  |  |  |  |  |
| **Age** | 46.33 (10.56) | 44.24 (10.96) | 18.36 (0.2) | F = 521.96, **p <.0001** | t = 1.58, p = 0.11 | t = 36.11, **p <.0001** | t = -24.18, **p <.0001** |
| **Sex (% male)** | 84% | 83% | 100% | X^2^ = 29.78, ***p* < .0001** | X^2^ = .01, *p* = .93 | X^2^ = 26.24, ***p* < .0001** | X^2^ = 26.95, ***p* < .0001** |
| **Years of education** | 14.85 (3.84) | 15.84 (3.24) | 11.65 (0.93) | F = 77.612, ***p* <.0001** | t = -2.28, **p = .02** | t = 10.75, **p <.001** | t = -12.67, **p < .0001** |
| **Clinical Characteristics** | |  |  |  |  |  |  |
| **Anxiety ^a^** | 4.44 (3.39) | 2.21 (2.08) | 2.46 (2.39) | F = 30.033, **p <.0001** | t = 6.83, **p < .0001** | t = 6.28, **p <.0001** | t = 0.88, p = 0.38 |
| **Depression ^b^** | 3.64 (3.64) | 1.57 (2.15) | 1.7 (1.81) | F = 27.724, **p <.0001** | t = 6, **p < .0001** | t = 6.31, **p < .0001** | t = 0.51, p = 0.61 |
| **Craving ^c^** | 12.72 (8.03) | 2.73 (2.93) | 3.77 (3.16) | F = 129.89, **p < .0001** | t = 14.31, **p < .0001** | t = 13.58, **p < .0001** | t = 2.46, **p = 0.02** |
| **Impulsivity ^d^** | 31.66 (6.48) | 29.2 (5.35) | 30.45 (5.06) | F = 6.12, **p = 0.002** | t = 3.45, **p = 0.001** | t = 1.93, p = 0.05 | t = 1.89, p = 0.06 |
| **Neuropsychological Testing** | |  |  |  |  |  |  |
| **Cognitive Speed ^e^** | 9.31 (2.76) | 10.67 (3.07) | 11.37 (2.31) | F = 25.78, **p <.0001** | t = -3.73, **p = 0.001** | t = -7.4965, **p <.0001** | t = 2.02, **p = 0.04** |
| **Working Memory ^f^** | 6.55 (1.87) | 7.46 (2.07) | 8.04 (2.01) | F = 24.60, **p <.0001** | t = -3.69, **p = 0.001** | t = -7.038, **p < .0001** | t = 2.26, **p = 0.03** |

# SI 4: Post-hoc analyses based on visual inspection of the 3-way interaction: Pavlovian valence x Instrumental action x OPRM1 polymorphism (analysis 1)

Interestingly, our graphical illustrations of the interaction between the PIT effect and the OPRM1 polymorphism (Figure S2) indicated that the direction of this association was group dependent. More precisely, Figure S2 suggests that AD G+ carriers showed increased modulation of Go and No-Go responses in the context of positive stimuli, whereas G+ carriers in both healthy control samples (middle aged controls and young controls) seemed to show increased modulation of Go and No-Go responses in the context of negative stimuli.

We thus performed exploratory post hoc analyses for all three cohorts, respectively, where we tested instrumental action*OPRM1 interactions separately for the negative (Negative vs. neutral) and positive (positive vs. neutral) limb of the PIT effect. In AD patients, we found a significant interaction between instrumental action and OPRM1 for positive values (p < .0001), but not for negative values (p = .29). In young and middle aged healthy controls, we found the reverse patterns, namely an instrumental action*OPRM1 interaction for negative values (young controls, p < .0001, middle aged controls, p < .0001) but no significant interaction between instrumental action and OPRM1 for positive values (young controls, p = .59, middle aged controls, p = .08).

# SI 5: Post-hoc analyses for the 3-way interaction: Instrumental action x group x OPRM1 polymorphism (analysis 2)

We performed additional post-hoc analyses for AD patients and healthy controls separately, where we analyzed how the OPRM1 polymorphism would differentially affect Go and No-Go responses. This analysis revealed that AD G+ carriers showed increased Go responses compared to No-Go responses compared to G- carriers p = .01) whereas healthy controls showed increased No-Go responses compared to Go responses compared to G- carriers (p < .0001).

# SI 6: Performance of the instrumental learning phase

448 out of the initial 452 subjects had complete data of the instrumental learning phase (phase 1 of the paradigm).

On average subjects performed 87.2 trials (sd = 25.7) until they reached the criterion in the instrumental learning phase. OPRM1 polymorphism did not covary with the number of trials that G- and G+ carriers performed (young controls: W = 2542.5, *p* = .74, middle-aged controls: W = 884.5, *p* = 0.48, AD patients: W = 2403.5, *p* = 0.91). Thus, overall performance in the instrumental learning phase was not dependent on OPRM1 polymorphism. Adapting our analysis approach from the PIT phase we also performed a binomial mixed effect regression, where we regressed instrumental action (Go, No-Go) and the OPRM1 polymorphism on accuracy (correct, incorrect) in the instrumental learning phase.

Overall, we found a main effect of instrumental action that was positive across all cohorts, indicating that subjects were better in Go learning compared to No-Go learning. Beyond this, the OPRM1 polymorphism did not interact with instrumental action in young healthy controls (*p* = .26), AD patients (*p* = .17), but was statistically significant in middle-aged healthy controls (*p* = <.0001). Post-hoc analyses indicated that in this cohort (middle-aged controls), G- carriers showed significantly better performance in Go compared to No-Go trials (*p* < .0001), whereas this difference was not significantly different in G+ carriers (*p* = .06), see Table S3, Figure S2.

Table S3: Results from the generalized linear mixed effects model of the instrumental training data

|  | **Alcohol-dependent patients (n = 184)** | | **Middle-aged controls (n = 103)** | | **Young controls (n =161)** | |
| --- | --- | --- | --- | --- | --- | --- |
|  | Estimate | p-value | Estimate | p-value | Estimate | p-value |
| Intercept | 0.59 | **<.0001** | 0.88 | **<.0001** | 1.11 | **<.0001** |
| Instrumental action | 0.48 | **<.001** | 0.74 | **<.0001** | 0.51 | **<.0001** |
| OPRM1 polymorphism | 0.08 | .39 | -0.36 | **.002** | -0.01 | 0.94 |
| Instrumental action * OPRM1 polymorphism | -0.11 | 0.17 | 0.57 | **<.0001** | 0.10 | 0.26 |


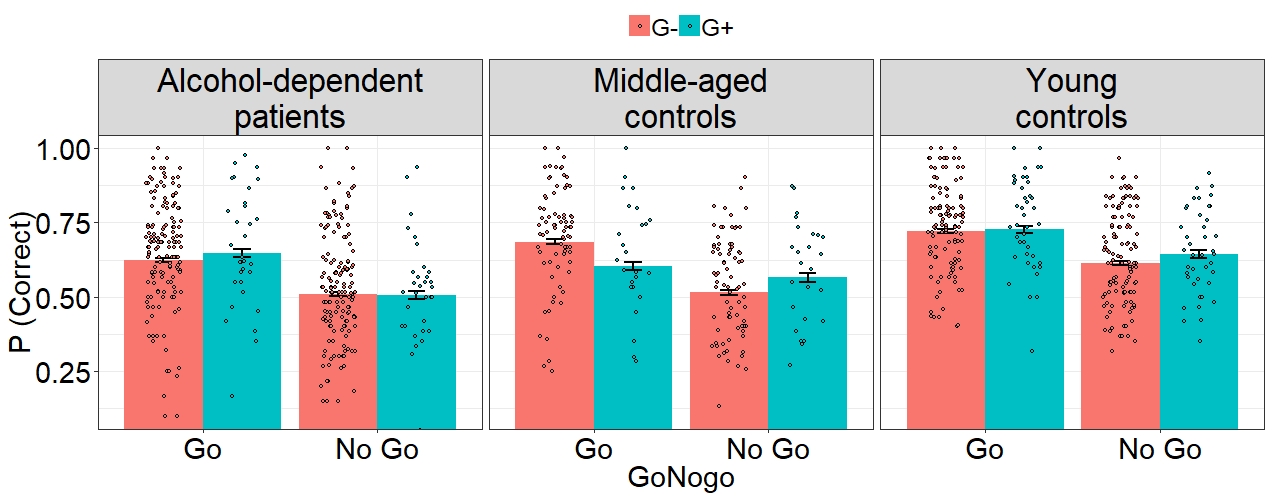


Figure S2: Correct responses in the instrumental learning phase as a function of action (Go/No-go), group (alcohol dependent, middle age controls, young controls) and OPRM1 polymorphism (G-/G+). For display purposes only responses larger than 10% correct are displayed.

# SI 7: Stay/switch behavior of the instrumental learning phase

Some previous studies have suggested a profound effect of the opioid system on reinforcement learning (Efremidze et al., 2017; Lee et al., 2011). Moreover, our analyses of the overall correct responses from the instrumental learning phase revealed that at least in one cohort (middle aged controls) Go and No-Go learning was different across G+ and G- carriers (see SI 5). Thus, we additionally tested, whether OPRM1 polymorphism additionally affected choice behavior depending on outcome. More precisely we asked, whether G+-carriers would show increased learning from reward or punishment. To this end, we carried out an analysis previously introduced by Huys et al. (2011), where we analyzed the immediate consequences of a trial’s outcome on subsequent behavior. We performed a binomial mixed effect regression, where we regressed outcome of the previous trial (reward_(t)_, punishment_(t)_) and the OPRM1 polymorphism on repetition behavior of a specific stimulus (stay = Go_(t)_ & Go_(t+1)_ | No-Go_(t)_ & No-Go_(t+1)_, switch = Go_(t)_ & No-Go_(t+1)_ | No-Go_(t)_ & Go_(t+1)_). This analysis revealed increased stay behavior after reward compared to switch behavior after punishment (see Figure S3, Table S4) across all cohorts. This is in line with Huys et al. (2011) and has previously been interpreted as increased reward sensitivity opposed to punishment sensitivity. OPRM1 polymorphism did not influence the impact that outcome of the previous trial (reward/punishment) had on stay/ switch behavior. However, in middle aged controls, the main effect of OPRM1 polymorphism was on the border of significance (*p* = .07), indicating that in this cohort, G+ carriers tended to show deficits in adapting their behavior according to the outcomes of their action, which is in line with the finding that in this cohort G+ carriers showed overall less correct responses in the instrumental learning phase (see Figure S 3, middle panel).

Table S4: Results from the generalized linear mixed effects model of the instrumental training data regarding stay/switch behavior as a function of previous outcome and OPRM1 polymorphism

|  | **Alcohol-dependent patients (n = 184)** | | **Middle-aged controls (n = 103)** | | **Young controls (n =161)** | |
| --- | --- | --- | --- | --- | --- | --- |
|  | Estimate | p-value | Estimate | p-value | Estimate | p-value |
| Intercept | 0.46 | **<.0001** | 0.42 | **<.0001** | 0.30 | **<.0001** |
| Reward/Punishment of the previous trial | 0.47 | **<.001** | 0.65 | **<.0001** | 0.91 | **<.0001** |
| OPRM1 polymorphism | 0.01 | .97 | -0.24 | 0.07 | -0.01 | 0.90 |
| Reward/Punishment * OPRM1 polymorphism | -0.03 | 0.69 | 0.02 | 0.82 | 0.15 | 0.12 |


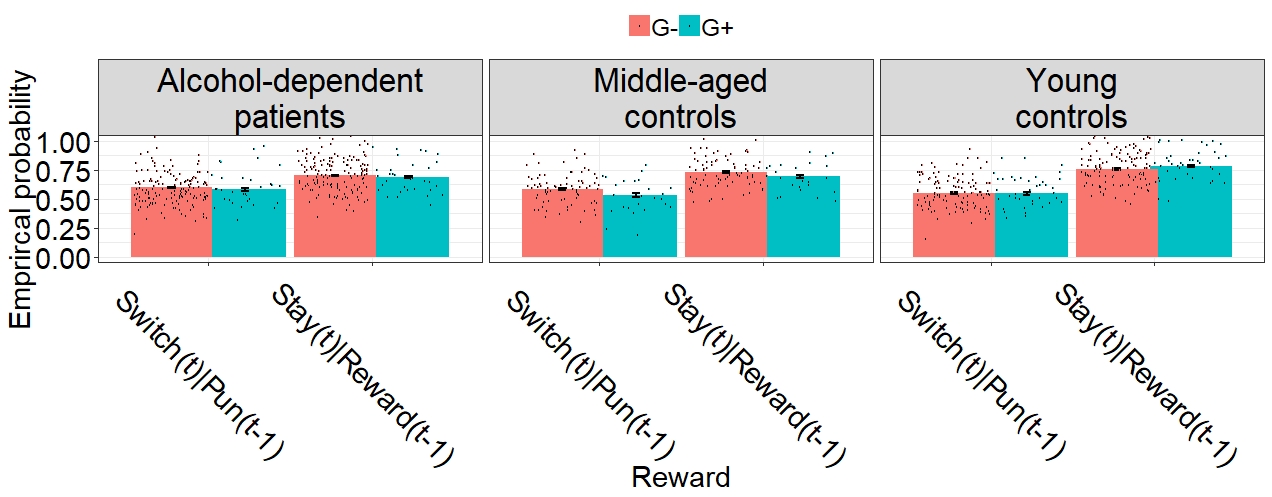


*Figure S3: Mean overall probability of repeating an action in the instrumental part given that it was last rewarded in the presence of the current stimulus, or the probability of switching given a previous punishment as a function of Group (Alcohol dependent, middle age controls, young controls) and OPRM1 polymorphism (G+/G- carriers).*

# SI 8: Performance of the forced choice phase

448 out of the initial 452 subjects had complete data of the forced choice phase (phase 4 of the paradigm). Based on prior work (Garbusow et al., 2016; Garbusow et al., 2014), we only included subjects in all analyses, who responded better than chance in the query trials (see SI 1). We did this to ensure that subjects had sufficiently acquired the Pavlovian associations, which is a prerequisite of the Pavlovian-to-instrumental transfer effect.

To mirror our behavioral analyses of the PIT phase and the instrumental learning phase, we analyzed performance from the query trials in the same way. Thus we regressed correct responses on Pavlovian valence by using a linear mixed model. Note that in each trial two Pavlovian values are displayed and subjects have to indicate the better one. Thus, if subjects give a correct response, this trial is coded as correctly for both Pavlovian values, whereas if the subject gives a wrong response, this trial is coded as false for both Pavlovian values. This results in 60 correct/false responses for each subject from 30 trials.

Results from this analysis indicated that Pavlovian valence influenced correct choices in AD patients (*p* < .0001) and in middle-aged controls (*p* < .0001) but not in young controls (*p* = .88, probably due to ceiling effects in this group). This effect was not modulated by the OPRM1 polymorphism in AD patients (*p* = 0.07), nor middle-aged controls (*p* = 0.16) or young controls (*p*= 0.8, Table S5, Figure S4)

Table S5: Results from the linear mixed effects model of the forced choice data

|  | **Alcohol-dependent patients (n = 184)** | | **Middle-aged controls (n = 103)** | | **Young controls (n =161)** | |
| --- | --- | --- | --- | --- | --- | --- |
|  | Chisq | p-value | Chisq | p-value | Chisq | p-value |
| Pavlovian valence | 46.24 | **<.0001** | 24.95 | **<.0001** | 0.26 | 0.88 |
| OPRM1 polymorphism | 2.56 | .11 | 0.92 | 0.34 | 0.38 | 0.54 |
| Pavlovian valence* OPRM1 polymorphism | 5.20 | 0.07 | 3.66 | 0.16 | 0.45 | 0.8 |


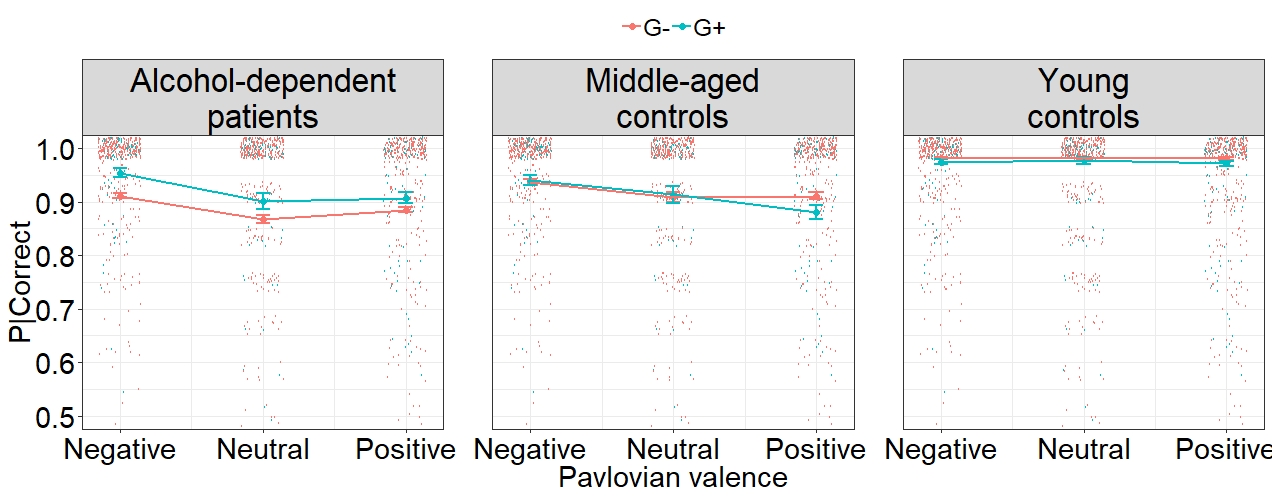


Figure S4: Correct responses for the forced choice task as a function of Pavlovian valence (Negative, Neutral, Positive) OPRM1 polymorphism (G-,G+ carriers) and group (alcohol-dependent, middle-age controls, young controls). For display purposes individual correct responses are clustered, in the way that subjects with the same correct responses for a Pavlovian valence are displayed as scatter.

# SI 9: Reaction times of the PIT phase

In the behavioral analyses, we did not see a OPRM1 polymorphism*Pavlovian valence interaction across all subjects, However, we found evidence that Pavlovian valence interacted more strongly with action across G+ carriers. We performed additional analyses to see how Pavlovian valence influenced reaction times.

Thus we regressed reaction times of first button presses on Pavlovian valence and OPRM1 polymorphism on reaction times of first button presses. First button responses were cleaned for reaction times faster than 50 milliseconds (0.006 % of all trials) or slower than 2950 ms (0.001% of all events). Note that even if a subject performs a response in a No-Go trial, this can be a correct response, because the coding of correct/ incorrect depends of the number of button presses. Thus we collapsed all trials across Go and No-Go responses.

Mirroring our behavioral analyses, we performed all analyses separately for the three groups. Our analyses indicated that across all groups, Pavlovian valence influenced RTs (Table S6, Figure S5). Moreover, in AD patients, G+ carriers showed a stronger modulation of Pavlovian valence than G- allele carriers. This result was mirrored by young controls, but it failed to reach statistically significance in middle aged healthy controls.

Table S6: Results from the linear mixed effects model of the Reaction time data of the first button press during the PIT phase

|  | **Alcohol-dependent patients (n = 186)** | | **Middle-aged controls (n = 105)** | | **Young controls (n =161)** | |
| --- | --- | --- | --- | --- | --- | --- |
|  | Chisq | p-value | Chisq | p-value | Estimate | p-value |
| Pavlovian valence | 342.19 | **<.0001** | 84.71 | **<.0001** | 45.22 | <.0001 |
| OPRM1 polymorphism | 0.01 | .93 | 3.36 | .07 | 0.18 | 0.67 |
| Pavlovian valence* OPRM1 polymorphism | 7.11 | **0.03** | 4.06 | 0.11 | 2.93 | **0.05** |


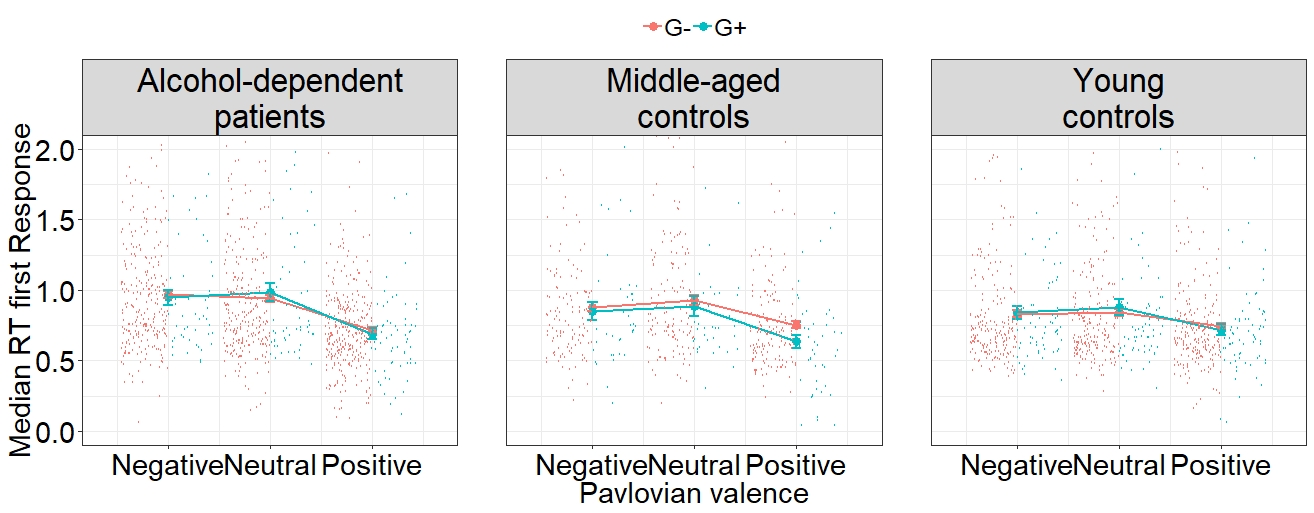


Figure S5: Reaction times of the first Response in the PIT phase as a function of Pavlovian valence (Negative, Neutral, Positive), OPRM1 polymorphism and group. For display purposes only individual responses < 2 seconds are displayed.

# SI 10: Subjective Pleasantness of the rating data

446 out of the initial 452 subjects had complete data of the rating part (phase 5 of the paradigm). We here additionally tested the hypothesis, that pleasantness ratings of instrumental and Pavlovian stimuli were affected by OPRM1 polymorphism across groups. With regard to the instrumental stimuli, we thus performed a linear mixed model, where we regressed OPRM1 polymorphism and instrumental action on pleasantness ratings. With regard to the Pavlovian stimuli, we performed a linear mixed model, where we regressed OPRM1 polymorphism and Pavlovian valence on pleasantness ratings.

Table S7: Results from the linear mixed effects model of the pleasantness ratings of the instrumental stimuli (above) and the Pavlovian stimuli (below).

|  | **Alcohol-dependent patients (n = 182)** | | **Middle-aged controls (n = 104)** | | **Young controls (n =160)** | |
| --- | --- | --- | --- | --- | --- | --- |
| *Instrumental stimuli* | Estimate | p-value | Estimate | p-value | Estimate | p-value |
| Intercept | 4.04 | **<.0001** | 4.08 | **<.0001** | 4.01 | **<.0001** |
| Instrumental action | 1.29 | **<.0001** | 2.13 | **<.0001** | 2.43 | **<.0001** |
| OPRM1 polymorphism | -0.10 | 0.53 | -0.14 | 0.48 | -0.01 | 0.94 |
| Instrumental action * OPRM1 polymorphism | -0.12 | 0.71 | -0.27 | 0.48 | -0.05 | 0.87 |
| *Pavlovian stimuli* | Chisq | p-value | Chisq | p-value | Chisq | p-value |
| Pavlovian valence | 127.95 | **<.0001** | 138.94 | **<.0001** | 311.84 | **<.0001** |
| OPRM1 polymorphism | 1.87 | 0.17 | 3.90 | 0.05 | 0.17 | 0.68 |
| Pavlovian valence * OPRM1 polymorphism | 3.06 | 0.22 | -0.13 | 0.24 | 2.52 | 0.28 |

With regard to the ratings of the instrumental stimuli, we found a main effect of instrumental action across groups (AD patients (*p* < .0001), middle aged controls (*p* < .0001) in young controls (*p* < .0001), demonstrating that subjects rated Go-trials as more pleasant compared to No-Go trials. Likewise, with regard to the ratings of the Pavlovian stimuli, we found a main effect of Pavlovian valence across groups (AD (*p* < .0001), middle aged controls (*p* < .0001) and in young controls (*p* < .0001), demonstrating that subjects rated the stimuli in accordance with their Pavlovian valence. However, neither ratings of instrumental nor Pavlovian stimuli were additionally modulated by OPRM1 polymorphism (Table S7 & Figure S6).


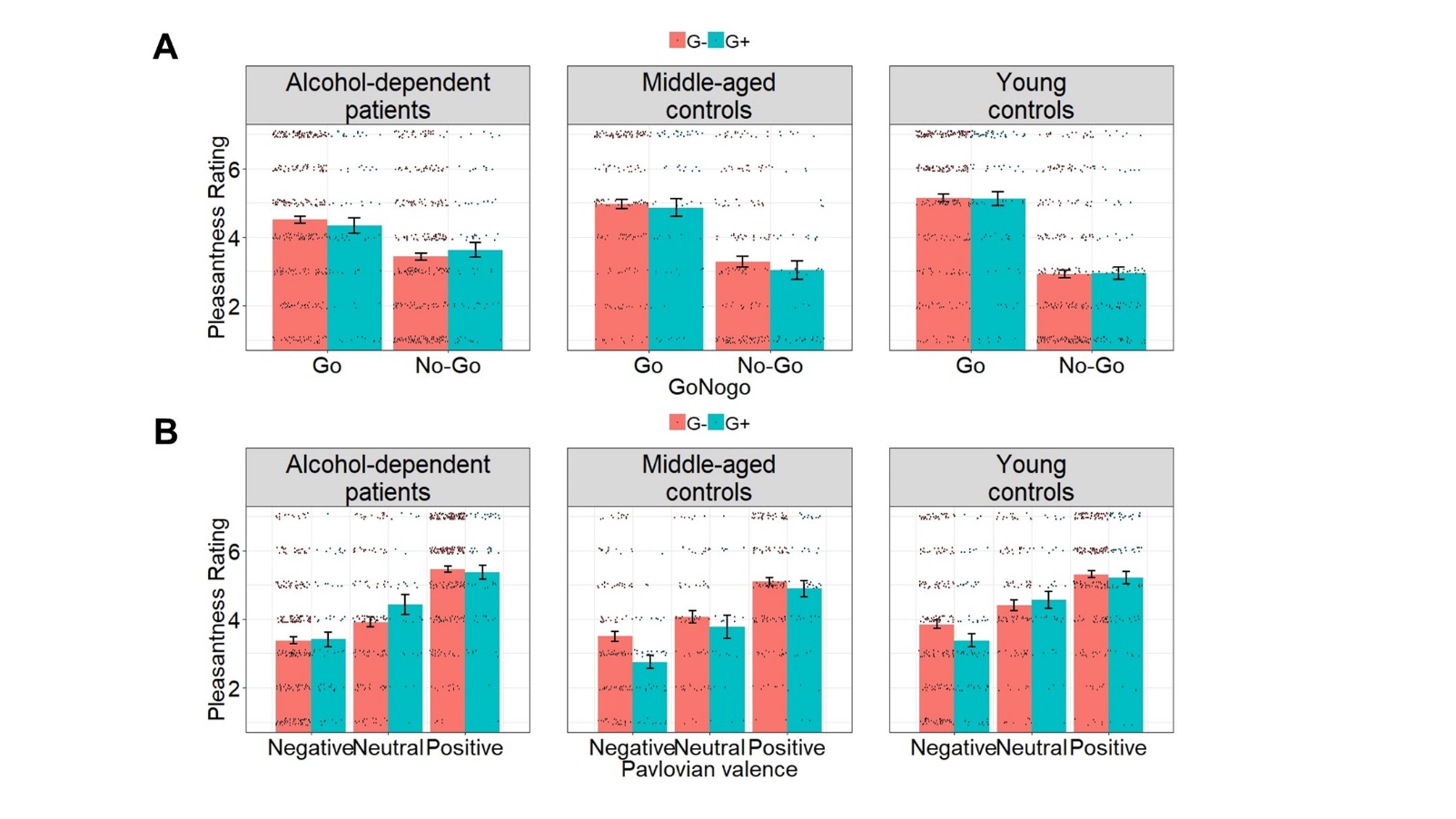


Figure S6: Pleasant ratings of **A.) Instrumental stimuli** and **B.) Pavlovian valence** as a function of OPRM1 polymorphism (G+,G- carriers) and group (Alcohol dependent, middle age controls, young controls)

# References

Efremidze L, Sarraf G, Miotto K, et al. (2017) The neural inhibition of learning increases asset market bubbles: Experimental evidence. *Journal of Behavioral Finance* 18: 114-124.

Garbusow M, Schad DJ, Sebold M, et al. (2016) Pavlovian-to-instrumental transfer effects in the nucleus accumbens relate to relapse in alcohol dependence. *Addict Biol* 21: 719-731.

Garbusow M, Schad DJ, Sommer C, et al. (2014) Pavlovian-to-instrumental transfer in alcohol dependence: a pilot study. *Neuropsychobiology* 70: 111-121.

Huys QJ, Cools R, Golzer M, et al. (2011) Disentangling the roles of approach, activation and valence in instrumental and pavlovian responding. *PLoS Comput Biol* 7: e1002028.

Lee MR, Gallen CL, Zhang X, et al. (2011) Functional polymorphism of the mu-opioid receptor gene (OPRM1) influences reinforcement learning in humans. *PLoS One* 6: e24203.

Sommer C, Garbusow M, Junger E, et al. (2017) Strong seduction: impulsivity and the impact of contextual cues on instrumental behavior in alcohol dependence. *Transl Psychiatry* 7: e1183.
